# Technical and natural conditions and operating efficiency of a municipal stormwater treatment plant

**DOI:** 10.1007/s11356-017-0519-8

**Published:** 2017-10-27

**Authors:** Tomasz Zubala

**Affiliations:** Department of Environmental Engineering and Geodesy, University of Life Sciences in Lublin, Leszczyńskiego 7, 20-069 Lublin, Poland

**Keywords:** Drainage, Environment, Pollutants, Stormwater

## Abstract

A decade of observations provided grounds for assessing the operation of one of the few stormwater treatment plants in Poland (system: screens—grit chambers—settler—retention pond) which collects effluents from 471 ha of the city. Among other aspects, the following were evaluated: treatment efficiency, relationship between the quality of treated stormwater and that of waters in the receiving body (the ox-bow lake of the Vistula river), operating stability of key units, significance of the facility for nature. During the assessment, the plant had a positive effect on the quality of stormwater effluents—the content of the analysed pollutants was reduced (more than 80% average efficiency for mineral forms of nitrogen and suspension matter) and oxygen ratios improved (23% increase in the average concentration of dissolved oxygen and more than 50% decrease in 5-day biochemical oxygen demand and chemical oxygen demand). Although the overall assessment of the facility’s operation was good, some omissions and operating errors were noted (method of removing retained pollutants, stormwater flow control). Eliminating them is a prerequisite for maintaining the expected reliability of the system. An effect of stormwater ponds on the increase in biodiversity in the poor urbanised landscape has also been observed. The structures, forming a uniform system along with urban green areas, constitute specific enclaves which attract living organisms.

## Introduction

One of the aspects of fast economic growth in many countries of Central and Eastern Europe is progressing urbanisation connected with the sealing, levelling and building development of large areas. As a consequence, the amount of stormwater in city catchments that must be collected and disposed of into receiving bodies is increasing. More and more often the unevenness of flow and hydraulic overload within sewage systems increases. A reduction in permeable surfaces in addition decreases the amount of water soaking into soil, and thus reduces the possibility of natural self-purification and renewal of underground water resources (Geiger and Dreiseitl 2001; Congying 2012; Zubala and Patro 2015). The stream of stormwater and melt-water can contain considerable amounts of pollutants, and so they pose a significant risk to natural receiving bodies (rivers, reservoirs) (Eriksson et al. 2007; Jamwal et al. 2008; Barałkiewicz et al. 2014). One of the most important methods of preventing overcharging of the sewage system and environmental pollution should be a reduction in the amount of stormwater and transported pollutants already at the point of origin. Sometimes simple solutions such as retention ponds (German and Svensson 2005; Moore and Hunt 2012; Herrmann and Yoshiyama 2014) or wetlands (Nzengy’a and Wishitemi 2001; Herrmann 2012; Howitt et al. 2014), where, among other processes, sedimentation and biological reduction of pollutants occur, are sufficient. Due to the limited availability of open space, small systems for the purification of stormwater dominate in towns and cities. They are situated point-wise, in the immediate vicinity of drained areas, e.g. roads, car parks, fuel stations, small housing estates. Such systems include separators, wells, settling tanks and infiltration basins (Geiger and Dreiseitl 2001; Langeveld et al. 2012; Fuchs et al. 2013; Tran and Kang 2013; Zubala and Patro 2015). The operating principle of such devices is very simple. Separators with settling tanks operate on the principle of the separation of substances with different densities, solely under the effect of the force of gravity. Heavy suspensions sediment on the bottom, and drops of light liquids float up towards the surface of the purified medium. In more complex structures (e.g. coalescence separators), the processes of adsorption and coalescence take place in addition to the effect of gravity. Rainwater settling tanks may have various structures and sizes (e.g. underground cement chambers, plastic containers and open earthen structures). They are used primarily for the protection of water receivers from suspensions. Settling tanks are used both as final purification devices and as an intermediate element in the technological system. Their operation consists in slowing down the flow of the liquid, which allows sedimentation of solid particles. Infiltration devices, whose function is the periodic retention of rainwaters with simultaneous drainage into the ground, are also an interesting solution. The purification of stormwater in such devices takes place in the sediment layer and in the infiltration substrate (suitable ground permeability is important here). Sometimes the relevant surfaces are planted with hydrophilic plants to enhance the effectiveness of purification.

Regrettably, facilities for the storage and treatment of stormwater are still not very common in many countries (Sakson et al. 2014; Hlavínek and Zelenakova 2015; Kordana and Słyś 2016). Planning and investment processes often neglect optional uses of stormwater for household, municipal and environment protection purposes, etc. (Barbosa et al. 2012; Cettner et al. 2014). In some large urbanised areas, raw stormwater is disposed of directly to surface waters and essentially pollutes them. Such practices are beyond reason and are inadmissible in the situation of the deteriorating condition of water resources.

For instance, mismanagement of water and sewage is one of the major reasons why Poland is classified among the main polluters of the Baltic Sea. There are concerns that given the present trends the Baltic ecosystem might soon become completely deteriorated (HELCOM 2007; Conley 2012). Thus, regardless of the share in the overall pollution, studies into and documentation of threats posed by stormwater and treatment options available in specific conditions is a task that should be undertaken urgently. Good promotion and implementation of efficient solutions in stormwater management in urbanised areas are necessary.

This work aims at evaluating the operation of an innovative stormwater treatment plant collecting effluents from 471 ha of an area of the town of Puławy. This is the only such structure in Poland. The unique character of this kind of system results from the combination of purely technical solutions (screen chambers, grit chambers, settler) with semi-natural elements (retention pond, ox-bow lake). Such big and complex urban systems are rarely analysed. The studies carried out since 2005 have aimed to determine, among other things, the quality of stormwater in respective units (grit chambers, settler, retention pond) and the effectiveness of its treatment. Based on field observations, an attempt was made to evaluate the basic technical and operating parameters after 20 years of operation of the treatment plant. The hypothetical high usability of the presented solution was verified based on the degree of elimination of pollutants, comparison of the quality of stormwater after treatment with the quality of water in the receiving body, and stable operation of the key equipment.

## Materials and methods

Puławy is located in south eastern Poland in the Lublin region. The town extends over an area of 50.6 km^2^ and has nearly 50 thousand inhabitants (SO 2014). The chemical industry, represented by the Nitrogen Works, is one of the industries developing locally. Puławy is intersected by the Vistula—the main river in Poland which flows into the Baltic Sea. The river is 1022 km long and the average flow at the mouth is 1080 m^3^ s^−1^ (CSO 2014). The average total annual precipitation rate in the region covered by the study is 571 mm. In a year, 165 days with precipitation are recorded with the maximum number occurring normally in July and the minimum in winter/spring. The average annual temperature is 8.0 °C, with the average temperature in January being − 2.9 °C and in July 18.5 °C. The duration of snow cover is 60 days and the period of vegetation lasts 220 days (average daily temperature > 5 °C) (Kaszewski 2008).

**Fig. 1:**
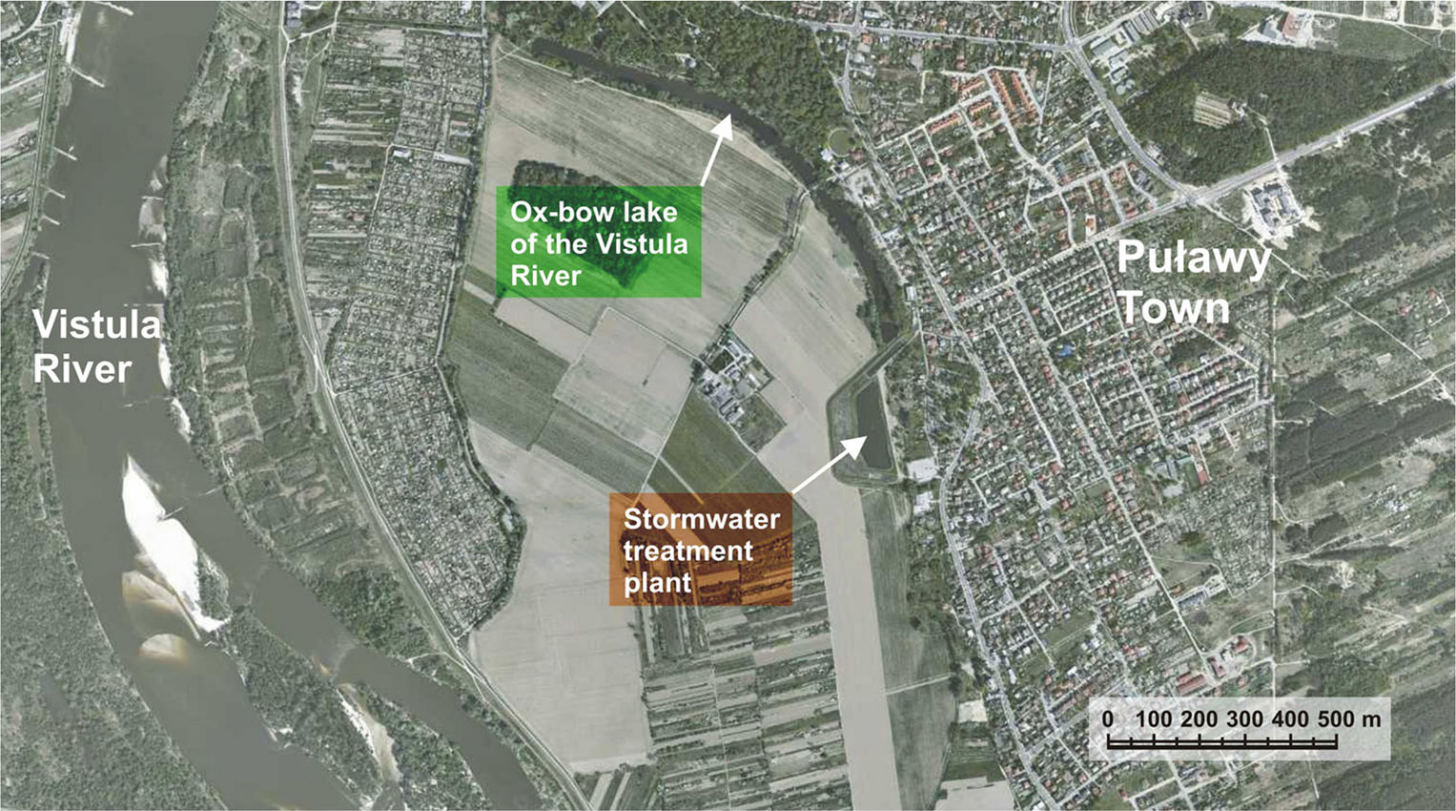
Location of the studied stormwater treatment plant (www.geoportal.gov.pl)

The stormwater treatment plant has operated in Puławy since 1995. It is situated in the lower flood plain of the Vistula at the end of the old riverbed (Fig. 1). It is surrounded by cropland of the Institute of Soil Science and Plant Cultivation in Puławy (on the river side) and poorly developed grounds with remains of old buildings (on the town side). A historic palace and park complex is located nearby with the above-mentioned old riverbed of the Vistula being an important element. The reservoir has an aesthetic and landscaping function (Patro and Zubala 2010).

**Fig. 2:**
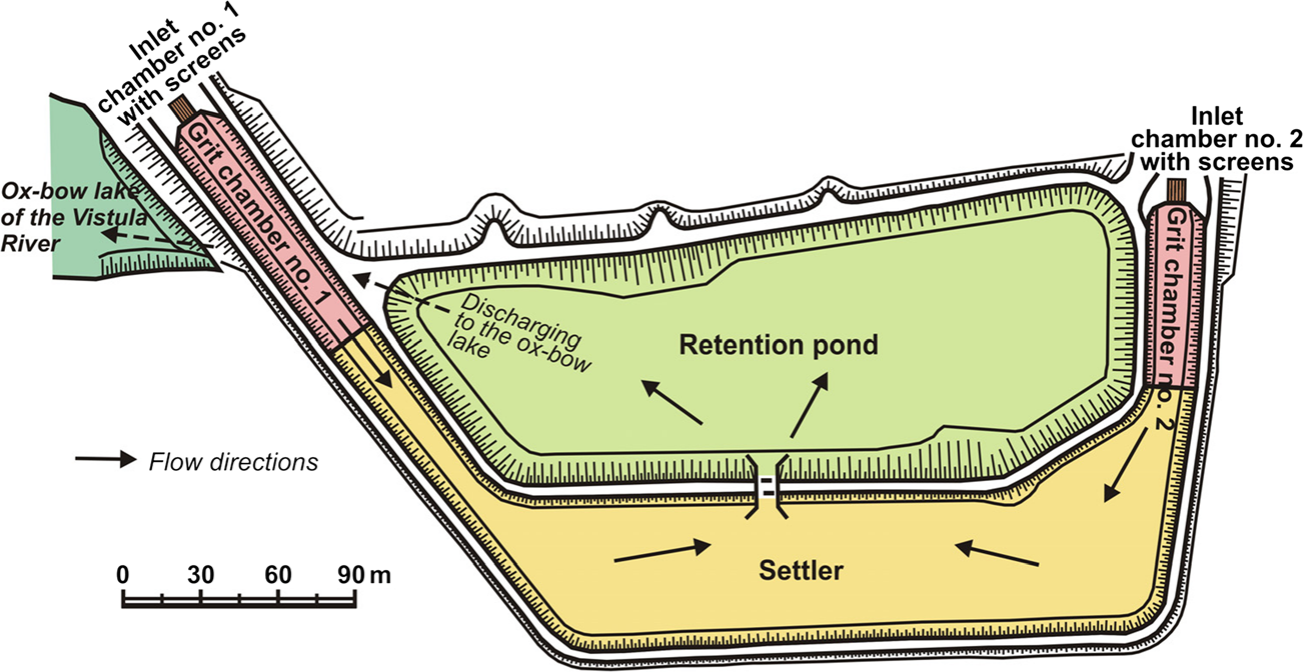
Scheme of the stormwater treatment plant in Puławy

In the analysed facility, stormwater is treated successively on screens, in grit chambers, settler and retention pond, and then carried away to the ox-bow lake of the Vistula (final receiving body) (Fig. 2). In terms of hydraulics, the system is based on the principle of “communicating vessels” and gravity flow. The core self-purification processes include straining, filtration, sedimentation, sorption, mixing, dilution, aeration and biological reactions. Most of these phenomena are common in natural and artificial water and marsh ecosystems (Dojlido 1995; Bratli et al. 1999; Ostroumov 2004; Dhote and Dixit 2007; Zubala 2009; Trowsdale and Simcock 2011; Tedoldi et al. 2016).

The municipal stormwater sewerage consists of two interceptors with the diameters of 1.4 and 1.6 m and smaller side sewers connected to the interceptors. The system is fitted with inspection chambers and street inlets. Interceptor no. 1 collects stormwater from the catchment with an area of 168 ha, covering the main street in Puławy and a large housing estate. Interceptor no. 2 transports stormwater from two housing estates including service, education and sports grounds. The catchment area of this interceptor is 303 ha. In a year with an average total rainfall, about 1 hm^3^ of effluents can be collected from the drained area.

**Fig. 3:**
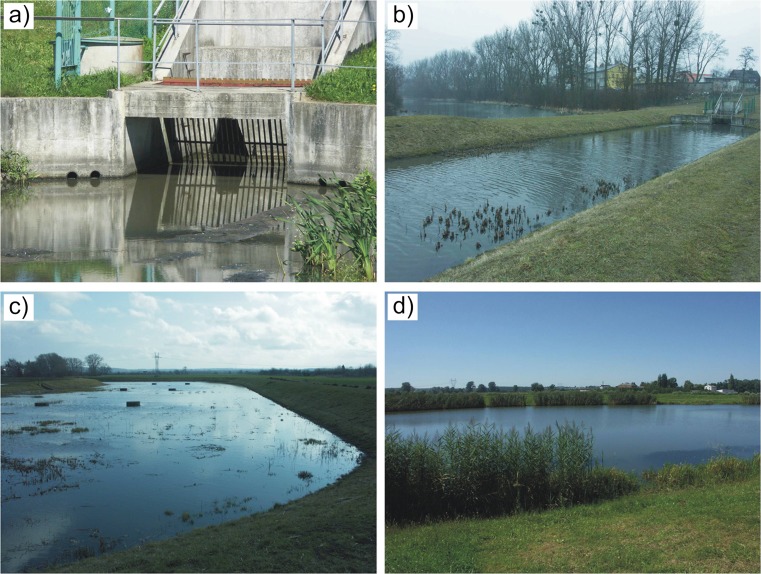
Basic units in the treatment plant: **a** screen in the inlet chamber 1; **b** grit chamber 1 (in the background the ox-bow lake of the Vistula—body receiving treated stormwater); **c** settler; **d** retention pond

In inlet chambers of the treatment plant (separate for both sewers) manually cleaned flat screens with an inclination of 60° and 8 cm clearance were installed (Fig. 3a). They also contain transverse concrete structures reducing the energy of received effluents.

When larger solid impurities are separated on the screens the stormwater flows into two grit chambers (separate for each inlet) which are 100 and 70 m long (Fig. 3b). The width of the grit chamber bottom is 10 m, working depth 1 m, slope 1:2. The design stormwater flow rate in grit chamber no. 1 is 0.42 m s^−1^, and in grit chamber no. 2–0.23 m s^−1^ (the time of keeping liquid in both grit chambers is similar). The parameters of the units facilitate sedimentation of a heavier fraction of mineral pollutants.

From grit chambers, stormwater flows through overflows with weirs to the settler where it is mixed (Fig. 3c). The main part of this structure is 190 m long and 46 m wide. The useful capacity is 12,540 m^3^ with a total area of 10,450 m^2^ and depth of 1.2 m. The settler mainly retains organic and light mineral suspension matter.

Next, stormwater is sent through a spillway with a diameter of 0.8 m (closed with weirs) to the retention pond—the last unit in the treatment plant (Fig. 3d). The usable area of the pond is 14,650 m^2^ and its total capacity 38,100 m^3^. The lower layer mainly supplied with ground water is maintained here—known as “biological water” (18,700 m^3^). The upper retention layer (maximum 19,400 m^3^) is carried away to the ox-bow lake of the Vistula by means of wells in the pond and in the ox-bow lake and spillways with a diameter of 0.4 m with weirs (two sewers under the bottom of grit chamber no. 1). The ordinates of the “biological water” level in the retention pond and that in the ox-bow lake of the Vistula neighbouring with the stormwater treatment plant are identical. The bottoms of the grit chambers and the settler are designed above this level due to the necessity to drain the retained sludge (also sludge from time to time pumped out from the pond).

The bottoms of grit chambers and the settler are strengthened by lattice-work reinforced concrete slabs placed on geotextile. The units are drained, which makes it possible to dry them quickly and then clear off the retained sludge. In addition, the drainage collects permeating water, which reduces the possibility of contaminating underground waters.

A retention pond is a typical earth structure. It lacks technical reinforcement and, similar to other units, is not fitted with a sealing layer. The slopes are overgrown with plants preventing the impact of waves on the water and supporting the process of biological purification (Fig. 3d). Hydrophilic plants also grow at the base of the dykes of the grit chambers and the settler.

On the cropland side, the treatment plant is enclosed by a surrounding trench which prevents possible waterboarding of adjacent grounds. On the side of the town, the trench is redundant with regard to the direct neighbourhood of the flood plain slope.

The distribution of strata in the soil profile should prevent possible penetration of pollutants to shallow underground waters. This is characteristic of alluvial soil—it contains, among other formations, soils with good sorptive properties (Turski et al. 2008). At the same time, no drinking water intakes were identified in the direct neighbourhood, so no special protective treatment is required.

The physical and chemical properties of stormwater in respective units of the stormwater treatment plant were analysed seasonally (28 measurement terms). Samples were taken using a bailer. During sampling, most units contained the treated stormwater. The exception was the settler, which at times was empty due to the small amount of rainfall or operating activities. The following measures were determined in the samples: temperature, electrolytic conductivity (by conductometry), pH (by potentiometry), total suspended solids (by drying and weighing), dissolved oxygen (O_2_), 5-day biochemical oxygen demand—BOD_5_ (by dilution), chemical oxygen demand—COD (by dichromate method), ammonia nitrogen (N-NH_4_^+^), nitrate nitrogen (N-NO_3_^−^), nitrite nitrogen (N-NO_2_^−^), phosphate phosphorus (P-PO_4_^−^), sulphates (SO_4_^−^), iron (Fe^+^), potassium (K^+^) and chlorides (Cl^−^) (photometric determination). Chemical components (e.g. biogenes) were determined by means of photometers: MPM 2010 (WTW) and LF 205 (Slandi). In the evaluation of the quality of stormwater, the extreme and mean values of the analysed ratios were determined for every checkpoint. The statistical variability of results was based on the standard deviation and the coefficient of variation. The non-parametric Wilcoxon test was used to compare the variable quality of raw stormwater in the grit chamber (arithmetic means of results from both chambers were taken into account) and treated water in the retention pond. The analysis made it possible to check the operating efficiency of the stormwater treatment plant.

## Results and discussion

Stormwater carried by sewerage into the analysed stormwater treatment plant was characterised by a relatively high variation in quality (Table 1). Due to the higher intensity of usage of catchment no. 1 (e.g. the presence of the main street with high traffic intensity), stormwater in grit chamber no. 1 was usually of worse quality than that in grit chamber no. 2. This was demonstrated by higher values of conductivity, N-NH_4_^+^, N-NO_3_^−^, N-NO_2_^−^, K^+^ and from time to time Cl^−^. At times, higher concentrations of total suspended solids were found in grit chamber no. 2, which was due to incorrectly secured building works within catchment no. 2 (predominant loess suspension). Many authors point to a relationship between land development and the quality of stormwater discharges (Kayhanian et al. 2003; Chang et al. 2004; Mangani et al. 2005; Liu et al. 2013; Czemiel Berndtsson 2014; Petrucci et al. 2014; Peng et al. 2016). Mallin et al. (2000) have shown that human development is considered to have significant environmental consequences. Development can also pose an increased human health risk (increased outflow of pathogenic bacteria from areas with significant impervious surface).

**Table 1:** Characteristic values of quality ratios of stormwater in grit chambers (1) and retention pond (2) in 2005–2011 (statistical important difference in quality variables was determined for *α* = 0.01—Wilcoxon test)

Variables	Control point	Minimal value	Maximum value	Average	Standard deviation	Variation coefficient	Important difference
Temperature (°C)	1^a^	2.5	28.5	13.1	7.9	60.6	–
	2	0.5	27.0	12.9	8.0	61.6	
Conductivity (μS cm^−1^)	1	139	1315	615	334.0	54.3	+
	2	84	708	230	131.0	56.9	
pH	1	7.4	10.5	–	0.6	7.6	–
	2	7.2	9.6	–	0.5	6.5	
Suspension (mg dm^−3^)	1	7	162	53	45.8	87.1	+
	2	1	44	8	8.9	116.1	
O_2_ (mg dm^−3^)	1	3.1	13.3	7.7	2.6	34.2	+
	2	5.9	13.6	9.5	2.0	21.1	
BOD_5_ (mg dm^−3^)	1	2.0	14.3	7.0	2.4	34.1	+
	2	0.8	4.9	2.9	1.1	36.4	
COD_Cr_ (mg dm^−3^)	1	15	86	43	18.5	42.9	+
	2	5	55	21	11.6	54.2	
N-NH_4_^+^ (mg dm^−3^)	1	0.055	25.310	2.181	4.7	216.0	+
	2	0.030	2.200	0.250	0.4	172.9	
N-NO_3_^−^ (mg dm^−3^)	1	0.113	5.944	1.391	1.6	111.6	+
	2	0.020	0.339	0.130	0.1	73.7	
N-NO_2_^−^ (mg dm^−3^)	1	0.015	0.347	0.124	0.07	59.9	+
	2	0.001	0.134	0.019	0.03	166.6	
P-PO_4_^−^ (mg dm^−3^)	1	0.075	1.630	0.417	0.3	80.3	+
	2	0.003	0.404	0.109	0.1	90.6	
SO_4_^−^ (mg dm^−3^)	1	5	104	31	24.5	79.2	+
	2	1	41	10	7.0	72.2	
Fe^+^ (mg dm^−3^)	1	0.3	3.1	1.1	0.6	58.0	+
	2	0.2	0.6	0.3	0.1	38.0	
K^+^ (mg dm^−3^)	1	2.3	38.5	14.1	8.8	62.6	+
	2	1.4	14.6	4.8	3.4	72.4	
Cl^−^ (mg dm^−3^)	1	6.8	160.0	42.5	34.5	81.1	+
	2	8.0	58.0	22.0	14.9	67.6	

^a^Mean value of quality variable for stormwater in grit chamber 1 and 2

Larger amounts of pollutants were received by the studied treatment plant during runoff after an extended rainless period. However, the load of pollutants tended to increase the most when snow melted. At that time, the conductivity in grit chambers was up to 1315 μS cm^−1^, the concentration of suspension reached 162 mg dm^−3^ and COD was at the maximum level of 86 mg dm^−3^. Also, an increased content of biogenic compounds was observed. Although the average concentration of chlorides in grit chambers did not exceed 42.5 mg dm^−3^, during the supply of salt used in the town to reduce slipperiness after snowfall their concentration amounted to 160 mg dm^−3^ (the contamination prevented the freezing of liquid in grit chambers even during frosty weather). According to literature, the content of Cl^−^ in raw melt-water effluents from urbanised areas can be several times higher (risk of salinity) (Sawicka-Siarkiewicz 2003; Corsi et al. 2015; Rivett et al. 2016). In cooler periods of the year, Cl^−^ was also recorded at slightly higher concentrations in the retention pond, which confirms that the units of the stormwater treatment plant have hydraulic connections.

Due to frequent oxygen deficits which can be observed in grit chambers, out of mineral forms of nitrogen N-NH_4_^+^ is predominant at this stage of treatment. Also, higher concentrations of P-PO_4_^−^ are found, most likely resulting from its additional release from bottom sediments (Braskerud et al. 2005). The level of concentration of O_2_ in the settler at some periods was also unsatisfactory. In the period of analysis, it ranged from 2.7 to 12.5 mg dm^−3^, with the mean value being 6.7 mg dm^−3^.

In colder terms (November–April), at nearly all times, the concentration of nutrients in the treated stormwater increased compared to the situation in warmer periods (May–October). For example, the average concentrations of N-NH_4_^+^ and N-NO_3_^−^ in the grit chambers were, respectively, 48.5 and 36.4% lower in the warmer period than in the colder season of the year. On the other hand, the concentration of the above-mentioned components in the retention pond decreased by 52.6 and 23.5% in the warm season of the year. During the vegetation period, autotrophs grew intensively, which was most likely connected with an increased assimilation of nitrogen from stormwater and sludge as well as a reduced flow of these pollutants from the basin (Arheimer et al. 1996; Jarvie et al. 1998; Birgand et al. 2007).

Decreased pollutant loads and smaller differences between them in respective units of the stormwater treatment plant were recorded after long-lasting rainfall. After most pollutants have been collected from atmospheric air and from the drained area of the town, at subsequent stages of rainfall clear liquid of relatively good quality entered the stormwater treatment plant (Lee et al. 2002; Liu et al. 2013). It has a diluting and aerating effect on stormwater from previous runoffs. In such conditions, even in grit chambers, conductivity amounted to less than 140 μS cm^−1^, the concentration of suspension matter did not exceed 10 mg dm^−3^, oxygen saturation reached 13.3 mg dm^−3^ and nutrient concentrations were the lowest of all recorded concentrations.

During observation, the stormwater treatment plant was shown to have a positive effect on the overall quality of stormwater. The content of pollutants was gradually reduced at subsequent stages of treatment (significance of differences proved by Wilcoxon test for the level of 0.01). This was particularly visible in the case of mineral compounds of nitrogen and total suspended solids where the average reduction rate exceeded 80% (the averaged rates in the grit chambers were compared with that in the retention pond). The degree of elimination of P-PO_4_^−^, Fe^+^, SO_4_^−^, and K^+^ was also satisfactory—depending on the component it ranged from 66 to 74% (Table 2).

**Table 2:** Percentage differences in average value of quality variables of stormwater at different stages of treatment (“-”—decrease, “+”—increase)

Variables	Stage of stormwater treatment		
	Grit chambers^a^—settler	Settler—retention pond	Grit chambers—retention pond
Temperature	− 10.8	+10.5	− 1.4
Conductivity	− 1.9	− 61.8	− 62.5
pH	− 3.0	+1.1	− 2.0
Suspension	− 59.2	− 64.3	− 85.5
O_2_	− 13.6	+ 42.8	+ 23.4
BOD_5_	− 9.5	− 54.3	− 58.6
COD_Cr_	− 9.8	− 44.7	− 50.2
N-NH_4_^+^	− 3.3	− 88.2	− 88.6
N-NO_3_^−^	− 59.7	− 76.8	− 90.7
N-NO_2_^−^	− 49.8	− 69.7	− 84.8
P-PO_4_^−^	− 25.6	− 64.9	− 73.9
SO_4_^−^	− 36.6	− 50.7	− 68.7
Fe^+^	− 24.0	− 63.7	− 72.4
K^+^	− 24.3	− 55.3	− 66.1
Cl^−^	− 0.7	− 47.8	− 48.2

^a^Mean value of quality variable for stormwater in grit chamber 1 and 2

The largest decrease in the content of pollutants was recorded between the settler and the retention pond—this referred to ratios such as, for example: N-NH_4_^+^ (88.2%), N-NO_3_^−^ (76.8%), and N-NO_2_^−^ (69.7%). One of the most important self-purification processes can be dilution in the lower “biological layer” (continuously maintained in the pond) supplied with filtered groundwater. A relatively high effectiveness of treatment could also be observed when comparing the grit chamber with the settler. The concentration of the suspension and N-NO_3_^−^ decreased by about 59% and that of N-NO_2_^−^ by nearly 50% (Table 2).

No distinct changes in purification effectiveness were noted with the passage of time. The phenomena described earlier recur in a cyclic manner in almost every year or season. Their scale, however, may differ slightly. Due to the very large number of natural and anthropogenic factors, the causes of the changes are difficult to determine. The decrease in the content of contaminants and increase in the concentration of O_2_ between successive devices retain constant trends. If a decrease of purification effectiveness was noted on a given date, it was usually related with a lower total load of contaminants inflowing to the treatment plant (lower differences of average content of contaminants in grit chambers and in retention pond). After the second year of observation, there was a considerable decrease in average electric conductivity in the retention pond (it usually did not exceed 200 μS cm^−1^, with grit chambers’ mean of 615 μS cm^−1^). In turn, in 2010 and 2011 (two final years of physical and chemical analyses) a slight increase was noted in the content of suspended solids (11.5 and 16.5 mg dm^−3^, respectively). On earlier dates of analyses, the content of suspension did not reach the level of 10 mg dm^−3^. Especially high average concentrations of suspension were recorded in grit chambers in 2009 and 2010 (approx. 73 mg dm^−3^). The smallest—23% reduction of COD_Cr_ was noted in the middle of the observation period (2008), and the highest—64.2%—in 2005. In the case of nutrients, the first year of the study proved to be exceptionally unfavourable, when the highest average concentrations were noted for N-NH_4_^+^ (grit chambers and retention pond), N-NO_3_^−^ and N-NO_2_^−^ (retention pond), P-PO_4_^−^ (grit chambers). In turn, the lowest average value of N-NH_4_^+^ was recorded in grit chambers in 2009, N-NO_3_^−^ and N-NO_2_^−^ in 2008, and P-PO_4_^−^ in 2010. The retention pond was decidedly the least contaminated with nutrients in the final year of analyses (N-NH_4_^+^, N-NO_3_^−^, P-PO_4_^−^).

Satisfactory oxygen conditions were observed in the case of stormwater collected in the retention pond (the final unit in the stormwater treatment plant). The average concentration of O_2_ was 9.5 mg dm^−3^ (the highest level of saturation was recorded in cold seasons), whereas BOD_5_ did not normally exceed 3 mg dm^−3^ (maximum value 4.9 mg dm^−3^) (Table 1). Compared to grit chambers, the average concentration of O_2_ increased by 23.4%, and BOD_5_ decreased by as much as 58.6%. This phenomenon is particularly significant for self-purification processes occurring in an aquatic environment (Braskerud et al. 2005; Juang et al. 2008). Also, a very low average conductivity in the retention pond is notable—several times lower than in natural surface waters (Dojlido 1995). In the entire stormwater treatment plant, the pH of the analysed stormwater was relatively high, ranging from 7.1 to 10.7. The periodic alkalisation of stormwater runoffs is likely to have been a result of the presence of alkaline ash, salinity and processes occurring in bottom sediments (e.g. stirred by intensive flows). Alkaline particles may originate from ash from household hearths and fireplaces, commonly used to sprinkle slippery road and pavement surfaces in winter.

No increased environmental pressure of the stormwater treatment plant was observed during the analyses. What is more, disposing of treated stormwater into the receiving body should improve its ecological conditions (e.g. dilution, aeration). Previous studies demonstrated that the quality of water in the ox-bow lake of the Vistula was very poor—this is an astatic reservoir with limited exchange of water, exposed to the risk of pollutants flowing from cropland and unkempt built-up land (Patro and Zubala 2010). In many cases, the waters of the ox-bow lake (receiving body) contained considerably more pollutants than treated rainwater. For example, the average BOD_5_ in treated stormwater was 186% lower compared to the ox-bow lake, whereas the concentrations of P-PO_4_^−^, Cl^−^, K^+^ and of the suspension were lower respectively by: 142, 112, 56 and 16%. The average content of mineral nitrogen compounds was comparable in both structures. Good quality of treated stormwater (taking only the analysed ratios into account) also makes it possible to use it for household purposes. Other authors have demonstrated that integrated management of stormwater can generate considerable economic benefits (savings on tap water), hydrological benefits (flood protection) and ecological benefits (protection of habitats) (Mitchell et al. 2007; Sharma et al. 2008; Yu et al. 2013; Tao et al. 2014; Arora and Reddy 2015; Pennino et al. 2016). In the conditions of the study, water from the retention pond can be used, e.g. for cleaning the large surface of the new car park (impermeable pavement). The object has an area of 0.4 ha and is situated in the immediate vicinity of the stormwater treatment plant. The used water will return to the screen chamber and grit chamber, thus creating a specific local closed circulation cycle. Purified stormwater can also be used for maintenance purposes at the nearby Roman Catholic cemetery (2 ha).

Despite the fact that the stormwater treatment plant is built in difficult geotechnical conditions and local soil is used as the building material, no excess displacements and deflections in respective structure and subsidence of adjacent land were recorded. No cracking or fissures were found in the body and the base of embankments. This may indicate the lack of intense infiltration of effluent from respective structures into the soil and dykes. Despite the high inclination of slopes, no erosion damage has been observed. Their surface is effectively protected by grass cover.

In open-air, units of the stormwater treatment plant organic deposits tend to accumulate from time to time. However, their regular removal (once every 1–2 years) prevents decay and thus reduces nuisances resulting from the emission of odours into the atmosphere.

**Fig. 4:**
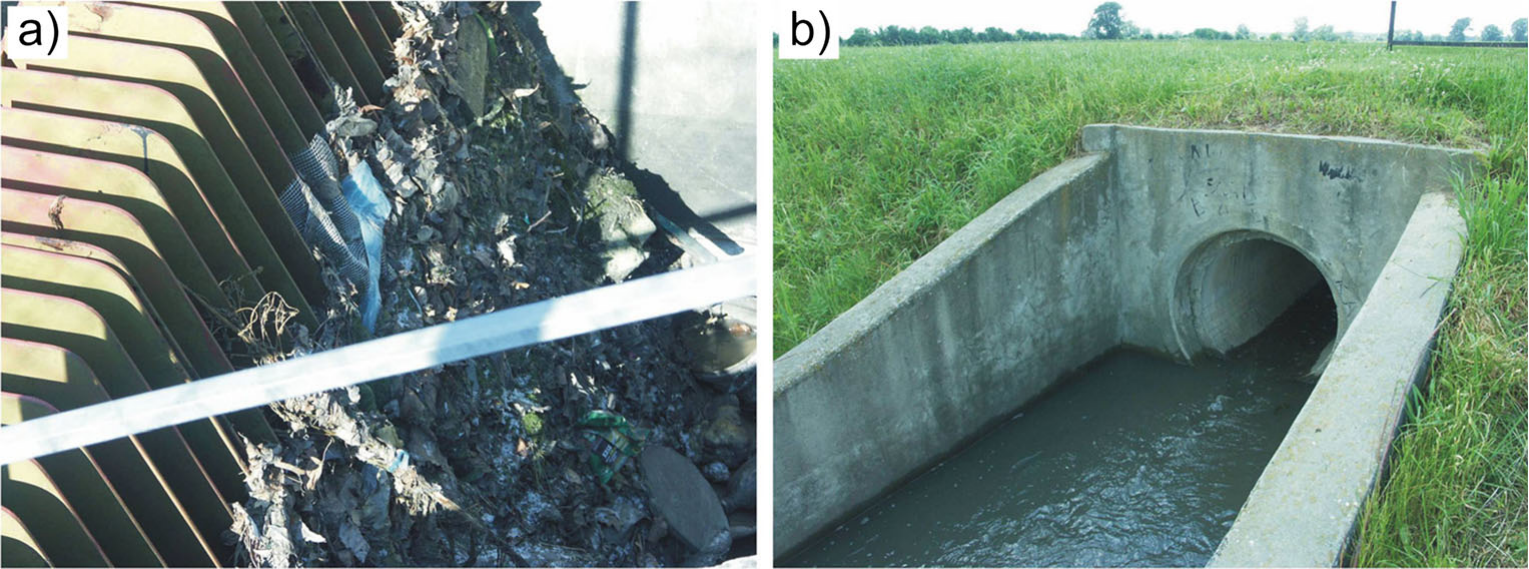
Examples of problems with the operation of the analysed treatment plant: **a** excessive amount of impurities deposited on the screen; **b** uncontrolled flow of stormwater from the settler to the retention pond

With certain exceptions, the basic rules of maintaining, operating and controlling the stormwater treatment plant are observed, which guarantees the proper functioning of respective units and of the whole facility and ensures the optimum stormwater treatment efficiency (Blecken et al. 2015). Omissions and errors which can cause short-term deterioration in the quality of the plant’s operation mainly concern the method of disposing of retained pollutants and controlling liquid flow. The problems are partly due to the highly random occurrence of rainwater supply and its quantitative and qualitative variability (Geiger and Dreiseitl 2001; Sawicka-Siarkiewicz 2003; Qin et al. 2010). The operation of screens should take into account more frequent disposal of waste accumulating on their surface (it should be performed after each intense supply preceded by a long rainless period). Waste deposited on the screens (Fig. 4a) makes the stormwater rise and causes uncontrolled sedimentation of mineral suspension in the sewer before the screen chamber. On the other hand, this is a way to observe improvement in the straining efficiency and related reduction in the content of suspension in stormwater in the subsequent unit (relatively high variability of the content of suspension in grit chambers).

Mechanical disposal of bottom sediments should be carried out more precisely—to prevent making changes in longitudinal profiles. The ordinate of the bottom of the settler cannot be higher than the ordinates of the bottom of the grit chambers. The removal of building material from the bottom of the grit chambers (lowering design ordinates) sometimes results in the stagnation of small amounts of stormwater in these units (problem of sedimentation of organic fraction). This was observed, among other periods, at the time when the settler was empty.

The discharge of stormwater from the settler into the retention pond should be better controlled. The weir must be lowered gradually and water should be disposed of in layers—to prevent the flow of liquid not completely clear of the bottom layer. During the analyses in some cases, the flow was improperly regulated (Fig. 4b), which at times deteriorated the quality of stormwater treated in the retention pond. A problem was, for example, dropping suspension which must be retained in the preceding units. For this reason, although the average quality of water treated in the retention pond is good, there is also a need to control the damming on the outflow to the ox-bow lake of the Vistula (e.g. extending the time of retention of the usable layer in the final reservoir of the plant).

If it is necessary to enhance the effect of treatment, the possibility of using one of the innovative solutions such as floating treatment wetlands should be considered. According to many authors, floating systems guarantee additional elimination of total nitrogen and phosphorus in a considerable range (efficiency is determined, among other factors, by the initial concentration of pollutants, species of plants used, temperature and season of the year) (Chang et al. 2012; Wang and Sample 2014; Wang et al. 2014).

**Fig. 5:**
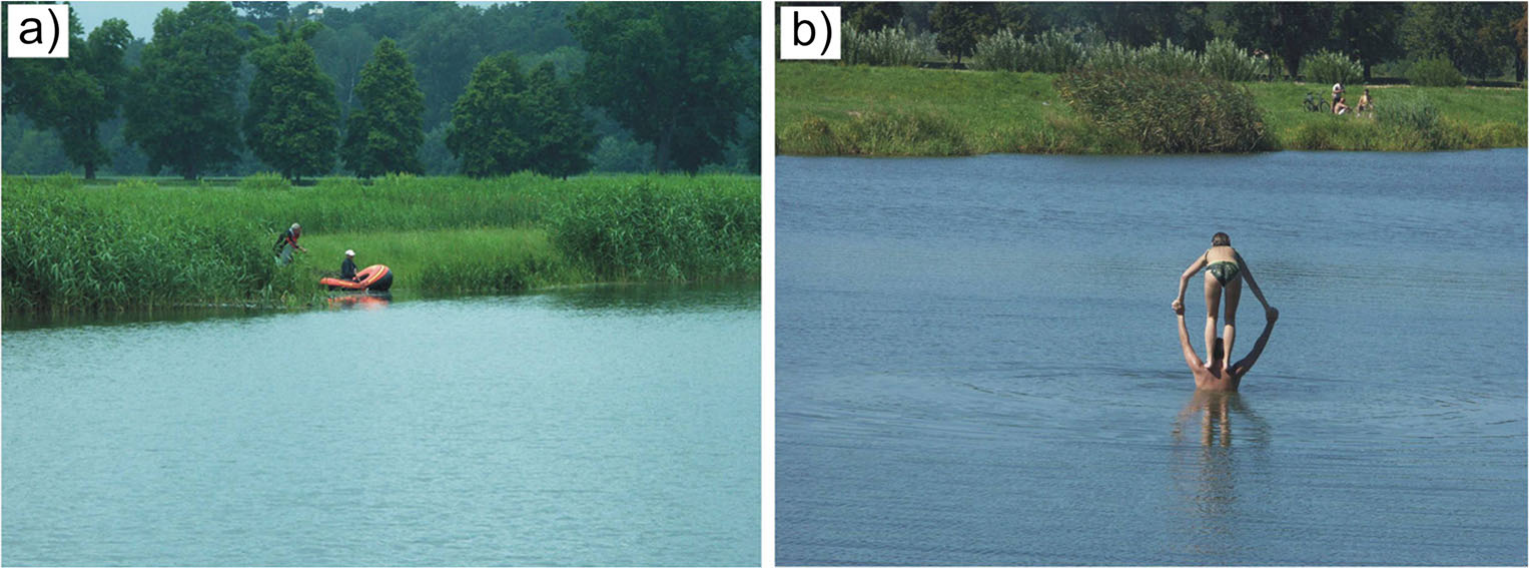
Attempts at illegal use of the stormwater treatment plant: **a** angling; **b** bathing

One of the priority tasks is to improve the level of security of the analysed facility and issue a ban on unauthorised access. The lack of effective restrictions and proper information policy is connected with the acts of devastation of certain units (e.g. moving elements of spillways). Also, unlawful attempts at using the premises of the stormwater treatment plant for leisure purposes constitute a serious problem. The uniqueness of such facilities and their attractive location encourages local inhabitants to walk and organise picnics within their premises and even use the reservoirs for fishing purposes (Fig. 5a). This lack of elementary knowledge and ecological awareness may be testified by the fact that the area of the treatment plant is treated as a bathing zone (Fig. 5b). Regrettably, in certain countries, it is still believed that stormwater (regardless of its origin) is always a natural and harmless component of the environment—resistant to negative anthropogenic impact. Here, a proper education process should play an important role (Baxter et al. 1985; Quigley and Taylor 2004; Taylor et al. 2007).

**Fig. 6:**
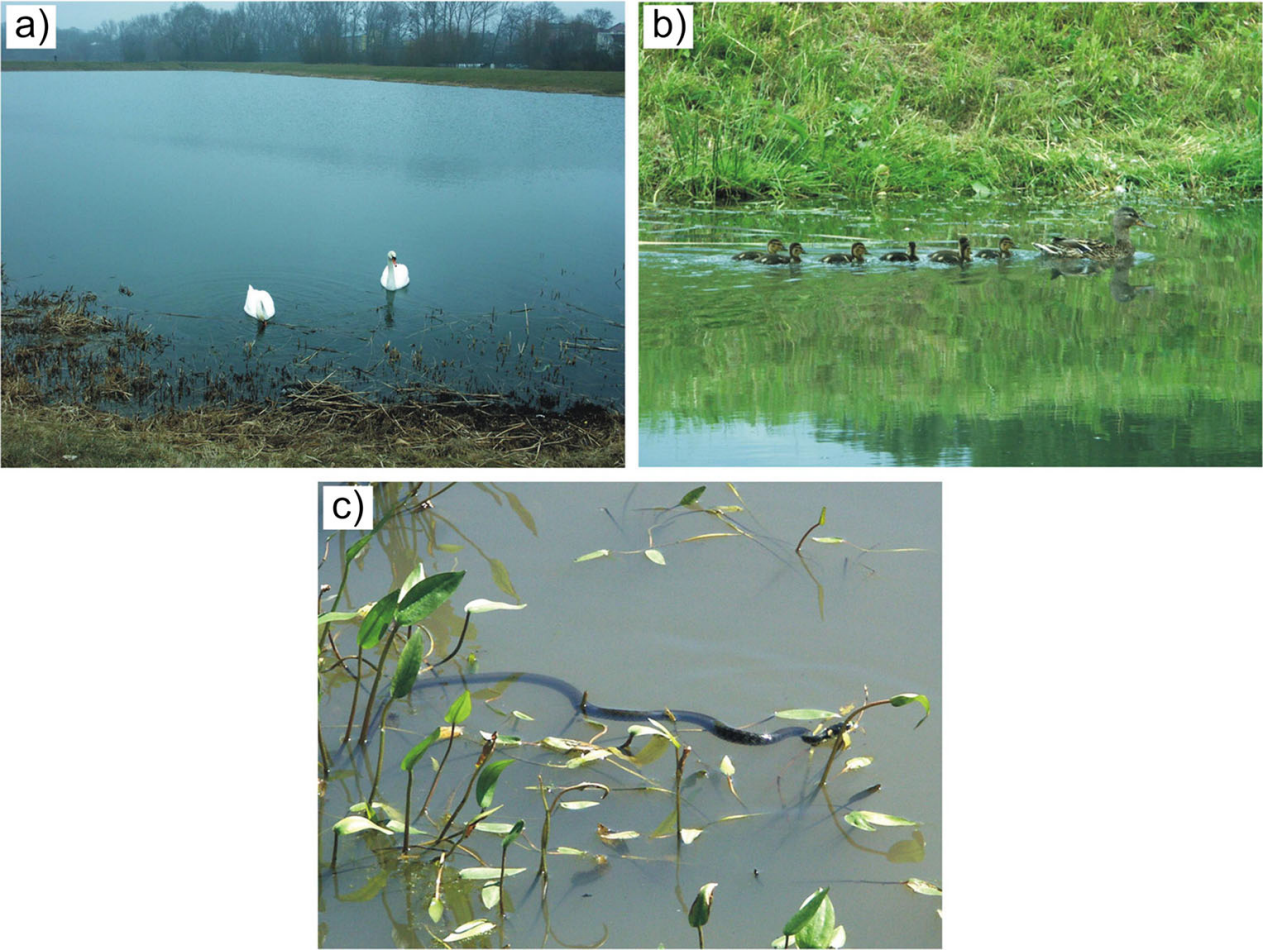
Examples of animals observed within the premises of the stormwater treatment plant: **a** mute swan *(Cygnus olor)*; **b** mallard *(Anas platyrhynchos)*; **c** grass snake *(Natrix natrix)*

On the other hand, stormwater reservoirs and wetlands can be a factor increasing biodiversity in a poor, urbanised landscape (Le Viol et al. 2009; Kazemi et al. 2011; Herrmann 2012; Zubala and Patro 2015). Spatial development schemes used for decades in the large urbanised areas of Central and Eastern Europe were mostly not conducive to the development or maintenance of biological life at an adequate level. The structures retaining rainwater, forming a uniform system along with urban green areas, could be a kind of enclave attracting living organisms which as a rule are not found in cities. In habitats characterised by varied trophy and moisture content the basic life needs could be satisfied more easily (improvement of living conditions). Within the analysed stormwater treatment plant in the studied period, intensive growth of water plants was observed—e.g.: common reed *(Phragmites communis)*, reed-mace *(Typha latifolia)* and rush *(Juncus inflexus)* (Fig. 3d), along with the presence of numerous representatives of fauna such as: the mute swan *(Cygnus olor)* (Fig. 6a), mallard *(Anas platyrhynchos)* (Fig. 6b), marsh harrier *(Circus aeruginosus)*, grass snake *(Natrix natrix)* (Fig. 6c), pond turtle *(Emys orbicularis)*, as well as certain species of amphibians, fish, molluscs and arthropods.

## Summary

According to surveys, a municipal rainwater management system consisting of typically technical and semi-natural elements is a highly effective solution. Efficient operation of the plant for most of the time during the observation testifies to the optimum selection of solutions in comparison to the existing conditions (e.g. adequate reinforcements and hydraulic parameters). Incidental complications can be partly explained with the highly random occurrence of rainwater supply and its variable quantity and quality. Insufficient knowledge of municipal services in charge of system operating can be of significance here. Unfortunately, there are also considerable delays in the environmental education process of local communities (unacceptable use of the facility for recreational and fishing purposes).

The high degree of elimination of pollution can be evidence for the correct functioning of the analysed system. The treatment efficiency was particularly high for mineral forms of nitrogen, total suspended solids, phosphate, iron and potassium. No clear changes were observed in the effects of treatment and with the lapse of time. The above-described phenomena recurred nearly every year or at a specific time of year. Factors that must be taken into account in such facilities include large increase in the load of pollutants during thaw and runoffs after a longer rainless period, or an increase in the concentration of nutrients in the treated rainwater in colder terms.

In the future, we should also consider the possibility of using treated rainwater for household purposes under the implementation of the concept of sustainable management of water resources. It is particularly important in the circumstances of the increasing “water stress” caused by a continuous increase in the needs and the number of users of the water resources as well as progressing climatic changes. The analysed system shows that the city can change its image and become more environment-friendly. In that respect, stormwater reservoirs also have landscaping functions since biological diversity may be increased in their region.
